# Architecture-Independent Absolute Solvation Free Energy Calculations with Neural Network Potentials

**DOI:** 10.1021/acs.jpclett.5c02980

**Published:** 2025-11-11

**Authors:** Anna Katharina Picha, Sara Tkaczyk, Thierry Langer, Marcus Wieder, Stefan Boresch

**Affiliations:** † 31285 University of Vienna, Faculty of Chemistry, Institute of Computational Biological Chemistry, 1090 Vienna, Austria; ‡ 27258 University of Vienna, Vienna Doctoral School of Chemistry (DosChem), 1090 Vienna, Austria; § Department of Pharmaceutical Sciences, Pharmaceutical Chemistry Division, Josef-Holaubek-Platz 2, University of Vienna, 1090 Vienna, Austria; ∥ Vienna Doctoral School of Pharmaceutical, Nutritional and Sport Sciences (PhaNuSpo), University of Vienna, 1090 Vienna, Austria; ⊥ Open Molecular Software Foundation, Davis, California 95616, United States

## Abstract

Allowing atoms or molecules to disappear is a critical step in alchemical free energy simulations (FES). The necessary tricks are well understood when using force fields. Over the past few years, neural network potentials (NNPs) have seen rapid development. Their potentially higher accuracy compared to force fields makes them attractive for use in FES. Here, we outline a method for gradually decoupling atoms and molecules in systems that are fully described by NNPs. Specifically, we show that manipulating the neighbor list is equivalent to using soft-core potentials in force-field-based FES. Since constructing the neighbor list is a central step, regardless of the NNP’s inner workings, our approach is agnostic to NNP architecture. We validate the correctness of our methodology by demonstrating cycle closure for a model problem and report solvation free energies obtained with the MACE-OFF23­(S/M) NNP.

## Graphical abstract

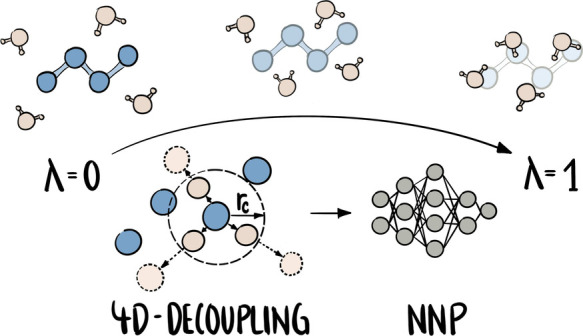

*Background*. Knowledge of the free energy difference (*ΔG*) provides information, in which direction a process or reaction will, in principle, occur spontaneously. Therefore, the calculation of *ΔG* by so-called free energy simulations (FES) has become an indispensable tool in the arsenal of computational chemists. Applications include the prediction of solvation free energy differences^1–3^ and partition coefficients.^3,4^ Of particular importance is the calculation of binding free energy differences that are now routinely used in lead optimization.^5,6^

The approximate nature of the molecular mechanical (MM) force fields (FF) used in the vast majority of FES limits the accuracy of the calculated *ΔΔG*s. The use of quantum chemical (QM) or hybrid QM/MM methods could, in principle, improve the accuracy, but FES with a QM/MM Hamiltonian are too slow for many applications of interest. Recent years have seen the rapid development of neural network potentials (NNP), sometimes also referred to as machine-learned interatomic potentials. These are neural networks trained on a large set of high-level quantum mechanical (QM) energies and forces and then used to obtain energies and forces with near QM accuracy at a fraction of the computational cost.^7,8^ A variety of NNP architectures have been developed during the past years, differing in their complexity, accuracy, and computational efficiency. Examples include AIMNet2,^9^ SchNet,^10^ PaiNN,^11^ NequIP,^12^ SpookyNet,^13^ ANI,^14,15^ MACE,^16^ among others. Similarly, a growing number of data sets have become available for training these models.^15,17–20^ In contrast to quantum chemistry, NNPs and hybrid NNP/MM approaches are sufficiently fast to simulate, e.g., solute–solvent or small protein–ligand systems for multiple nanoseconds. Their potentially higher accuracy compared to FFs makes the use of NNPs (or NNP/MM) in FES desirable.

*Challenges of Alchemical Transformations Using NNPs*. Similar to QM­(/MM), it is an open challenge how to perform the nonphysical transformations required by alchemical FES when using NNPs (NNP/MM). FES rely on nonphysical intermediate states and pathways. With FFs, it is possible to have, e.g., a carbon with three or five bonds, and by using soft-core potentials^5^ one can gradually remove the interactions of one or more atoms with the rest of the system. QM Hamiltonians are much more “picky”; a tri- or pentavalent carbon is simply impossible, and NNPs have similar restrictions. When QM/MM is used in FES, these limitations are usually avoided by so-called *indirect* approaches,^21–23^ and these have also been used with NNP/MM.^24–26^ Nevertheless, it would be highly convenient if alchemical transformations using NNPs could be carried out *directly*. Moore et al. recently modified the MACE NNP to achieve alchemical transformations;^27^ see also Nam et al.^28^ Similar methodology has been described for the FeNNix-Bio1 foundation model.^29^ While these approaches are, in principle, applicable to other NNPs, they rely on NNP-architecture-specific modifications. The alchemical transfer method can be used with NNPs,^30^ but it requires larger system sizes, and it is not suitable to calculate absolute solvation free energy (ASFE) differences.

A central step of any alchemical transformation is the “decoupling” or “annihilation” of one or more atoms. E.g., to compute an ASFE, one has to turn off the interactions between solute and solvent while avoiding the so-called van der Waals end point catastrophe.^31^ Here we outline an approach based on the manipulation of the neighbor list. Since neighbor lists are required in any NNP, our approach is, in principle, independent of the architectural details of the network used. Below, we describe the theoretical and practical details of our methodology and provide first results of ASFE calculations. One should keep in mind that the performance of current NNPs outside their training domain, most often single-molecule properties, is still unreliable.^32,33^ Agreement of the calculated solvation free energies with experiment, therefore, is not a reliable indicator of correctness. Instead, we focus on cycle closure of relative and absolute solvation free energy differences between pairs of solutes for validation. A brief summary describing our approach to carrying out the alchemical transformations for relative solvation free energies (RSFE) is given in the Appendix.^34^

*The Central Role of the Neighbor List*. All NNPs require a neighbor list as the first step of the energy (and force) evaluation. The construction of this list, which includes only atom pairs *i*,*j* with interatomic distances *d*_*ij*_ < *r*_*cut*_, is sketched in Figure 1: E.g., for atom D of the hypothetical solute, only solute atoms C and B, water 4, and one of the hydrogens of water 2 lie within the cutoff distance *r*_*cut*_ (dashed circle).

**Figure 1:**
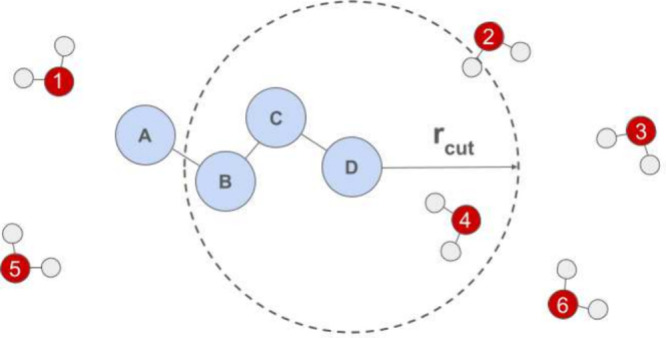
Hypothetical solute in water. The construction of the neighbor list is sketched.

Neighbor lists are also used in FF calculations, but in the case of NNPs, they need to contain more information. In particular, the distance *d*_*ij*_ for each atom pair *i,j* is tabulated for later use by the NNP. One immediate application, therefore, is the ability to exclude one (or more) atom(s) from the NNP energy/force evaluation. If one does not generate entries involving a particular atom in the neighbor list, then this atom is completely masked from the NNP. Such masking is central to our approach to carrying out relative alchemical transformations;^34^ see also the Appendix.

*4D-Decoupling*. In absolute solvation and binding free energy calculations, the interactions of the solute/ligand with its environment must be removed. In FF-based FES, soft-core potentials help to avoid end point problems.^5^ An alternative sometimes used with FFs is to add a “4D offset” to the interatomic distances;^35–37^ this is the approach we adapt to NNPs. Suppose we want to decouple the interactions of the hypothetical solute shown in Figure 1 with water. As one sees, water 4 and one hydrogen of water 2 are within the cutoff distance of solute atom D. To gradually remove the interactions of D with the solvent, we add an offset depending on the coupling parameter λ to the actual interatomic distance. Specifically, we replace the true distance *d*_*Dj*_ between atom D and water atoms *j* lying within *r*_*cut*_ by *d*_*Dj*_(λ) = *f*(*d*_*Dj*_, *r*_*cut*_, λ). The shifting function *f* can, e.g., be a linear offset or the Euclidean norm of a 4-dimensional vector and depends on the three parameters *d*_*Dj*_, *r*_*cut*_, and λ; for more details, see below. At λ = 0, the distances are unmodified, i.e., *d*_*Dj*_(λ = 0) = *d*_*Dj*_, while at λ = 1, *d*_*Dj*_(λ = 1) > *r*_*cut*_, i.e., all interatomic distances involving atom D are greater than *r*_*cut*_ and, therefore, entries for D and waters are not included in the neighbor list. The same is done (simultaneously) for the distances involving the other solute atoms, A, B, and C. Note that intramolecular distances, such as *d*_*CD*_, *d*_*BD*_ etc. are not modified; i.e., all intrasolute interactions remain unchanged. Similarly, neither intra- nor intermolecular solvent–solvent interactions are altered. Since all NNPs require the generation of a neighbor list, the “4D-Decoupling” just described is universally applicable and, therefore, independent of the architectural details of a particular NNP.

*Validation Strategy*. The 4D-Decoupling approach just outlined makes it possible to compute ASFEs when using NNPs to describe interactions. In addition, we know how to compute RSFE differences between tautomer pairs,^34^ i.e., two molecules that differ only in the location of a single hydrogen atom; see the Appendix. This makes it possible to compute the RSFE difference between two tautomers, T1 and T2, along two routes: (1) as the difference between the two ASFEs *ΔΔG*_*solv*_ = *ΔG*_*solv*_(*T*2) – *ΔG*_*solv*_(*T*1), and (2) using alchemical transformations, *ΔΔG*_*solv*_ = *ΔG* ^*aq*^(*T*1 → *T*2) – *ΔG* ^*gas*^(*T*1 → *T*2). The *ΔΔG*_*solv*_ values obtained according to (1) and (2) must agree within statistical error bars. This allows us to check their correctness self-consistently, *without* comparison with experiment. This validation strategy is analogous to the one we used to check for the correct treatment of dummy atoms.^38^

*Exploration of λ-Dependent Shifting Method*s. We considered four different λ-shifting schemes, denoted as *d*_*ij*_^(*k*)^(λ) where *k*∈{1, 2, 3, 4}, for manipulating interatomic distances *d*_*ij*_. All four schemes involve the atom-pair distance *d*_*ij*_, the neural network potential cutoff radius *r*_*cut*_, and the alchemical descriptor λ. They are designed to selectively modify the interatomic distances such that specific interactions, i.e., solute–solvent interactions for ASFE calculations, can be systematically “turned off” or “turned on”. At λ = 1, all interactions are nullified by ensuring that the modified distance satisfies *d*_*ij*_^(*k*)^(λ = 1) > *r*_*cut*_. Conversely, at λ = 0, all distances remain unmodified; i.e., *d*_*ij*_^(*k*)^(λ = 0) = *d*_*ij*_. The four schemes are divided into two main categories: linear shifting and 4D shifting. The linear shifting schemes (eqs 1a and 1b) correspond to a linear offset applied to the pairwise distances. The 4D shifting schemes (eqs 1c and 1d) use the Euclidean norm of a 4D vector, more closely resembling previously described 4D-decoupling methods.^35–37^ Within both the linear and 4D shifting categories, we implemented two distinct modification strategies: one that modifies distances by shifting atoms beyond the cutoff radius ([Disp-formula eq1a] and 1c), and another that shifts atoms precisely to the edge of the cutoff sphere (eqs 1b and 1d). These two approaches are visualized in Figure 2.

**Figure 2:**
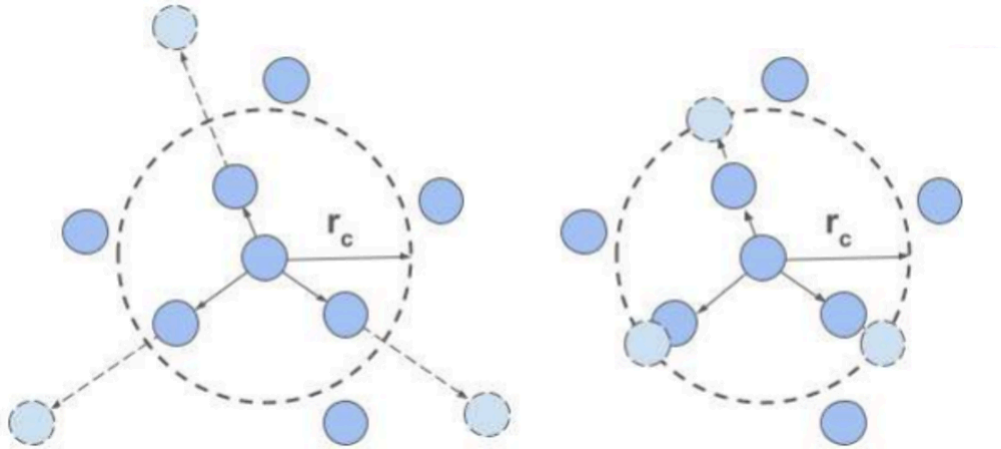
Left: “Naive” shifting by cutoff; see eqs 1a and 1c. Right: Shifting *to* cutoff; see eqs 1b and 1d.

*Linear Shifting Schemes*. The first scheme modifies distances so that *d*_*ij*_ is shifted by the cutoff of the NNP at λ = 1; i.e., *d*_*ij*_^(1)^(λ = 1) = *d*_*ij*_+*r*_*cut*_, making sure that all interactions are turned off. For an arbitrary λ ∈[0,1], the real distances *d*_*ij*_ between two atoms *i* and *j* are shifted as follows:

1a $$d_{ij}^{\left(1\right)}\left(\lambda \right)=d_{ij}+\lambda \cdot r_{cut}$$

The second scheme modifies distances so that *d*_*ij*_ is shifted exactly to the cutoff at λ = 1 (plus ϵ > 0, chosen based on the machine precision used, to account for rounding errors). Unlike the first scheme, the central atom interacts with all atoms within the cutoff radius at every λ-state, except at λ = 1, where interactions between atoms *i* and *j* are turned off:

1b $$d_{ij}^{\left(2\right)}\left(\lambda \right)=d_{ij}+\lambda \cdot \max\left(0,r_{cut}-d_{ij}\right)+\epsilon$$

*4D Shifting Schemes*. For an arbitrary vector *x⃗* = (*x*_1_, *x*_2_, ..., *x*_*n*_)^*T*^, the Euclidean norm is given by ${∥\mathrm{x⃗}∥}_{2}=\sqrt{\overset{n}{\underset{i=1}{\sum }}x_{i}^{2}}$ . That is, for a 3-dimensional vector *x⃗*_*ij*_ = *x*_*j*_–*x*_*i*_, ∥*x⃗*_*ij*_∥= *d*_*ij*_ denotes the length of the vector, i.e., the distance between atoms *i* and *j*. Let *x⃗*_ij_^*^ = (*x⃗*_*ij*_^*T*^, λ·*r*_*cut*_)^*T*^, so *x⃗*_ij_^*^ is a 4-dimensional vector where the first three components are equal to the components of *x⃗*_*ij*_. Then

$${∥{\mathrm{x⃗}}_{ij}^{*}∥}_{2}=\sqrt{x_{\mathrm{ij},1}^{2}+x_{\mathrm{ij},2}^{2}+x_{\mathrm{ij},3}^{2}+{\left(\lambda \cdot r_{cut}\right)}^{2}}=\sqrt{d_{ij}^{2}+{\left(\lambda \cdot r_{cut}\right)}^{2}}$$

Correspondingly, the first of the two 4D shifting schemes is given by

1c $$d_{ij}^{\left(3\right)}\left(\lambda \right)=\sqrt{d_{ij}^{2}+{\left(\lambda \cdot r_{cut}\right)}^{2}}$$

In analogy to eq 1b, our fourth approach *d*_*ij*_^(4)^(λ) is constructed so that *d*_*ij*_^(4)^(1) = *r*_*cut*_. ϵ is again added to take rounding errors into account:

1d $$d_{ij}^{\left(4\right)}\left(\lambda \right)=\sqrt{d_{ij}^{2}+\lambda \cdot \max\left(r_{cut}^{2}-d_{ij}^{2},0\right)}+\epsilon$$

*Simulation Details*. All calculations were carried out using OpenMM^46^ because of its support for NNPs and mixed NNP/MM systems.^39^ In particular, we used OpenMM’s coupling to MACE^47^ and the pretrained MACE-OFF23­(S) and MACE-OFF23­(M) models.^16^ All results reported below used single-precision (float32) arithmetic^48^ and were obtained with the “linear to cutoff” shifting scheme (eq 1b). For the small molecule solvation free energies, the following set of 15 λ-values: λ ∈ { $\frac{i}{19}$ |*i* = 0, 1, 2, 3, 4, 5, 6, 7, 8, 9, 11, 13, 15, 17, 19} was used; for the tautomer cycle closure calculations, see the Appendix. Additional results obtained with the other shifting schemes and the steps leading to the particular choice of λ-values are reported in the Supporting Information (SI).

For each solute, a simulation box was generated using pdbfixer^49^ with an initial box length of 24 Å, yielding simulation boxes containing 437–444 water molecules (see SI Table 2). Then, the boxes were equilibrated for 100 ps using the MACE-OFF23­(S) potential in single precision, shrinking the boxes to ≈23 Å. The resulting box size for each system is also listed in SI Table 2. Based on recent results using the MACE-OFF23­(S/M) models to study bulk water and other neat liquids,^33^ 100 ps of equilibration should be sufficient for the dilute solutions and small, rigid solutes considered here. At each λ-value, a 1 ns Langevin dynamics simulation with a Monte Carlo barostat was carried out at 300 K and 1 atm. Coordinates were saved at every 0.25 ps; the first 20% of each simulation were discarded as additional, λ-specific equilibration. Only every fourth of the saved coordinate sets (i.e., 800 per λ-state) were used for the actual free energy calculation (see below).

All simulations were initially carried out with a 1 fs time-step. Occasionally, simulations crashed using this setting. Upon investigation, we found that the instabilities were caused by spontaneous deprotonation events of either the solute or a water molecule. These runs were repeated with a time-step of 0.5 fs, leading to stable simulations. Saving frequencies, etc., were doubled in these cases. Each free energy difference was computed as the mean of three repeats; the standard deviation of the three individual *ΔG* results is used as the error estimate. For additional details concerning the RSFE (and ASFE) simulations of the tautomer pairs to test for cycle closure, see the Appendix.

*Practical Evaluation of Free Energy Differences*. Normally, we use MBAR to calculate free energy differences,^40^ specifically the pymbar implementation;^50^ e.g., for the calculation of relative free energy differences as described in the Appendix.^34^ However, when applied to data generated with the 4D-offset to compute ASFEs, pymbar would often fail to converge. Furthermore, MBAR results for the four λ-shifting schemes (see above) differed systematically. To investigate this further, we examined several configurations sampled at or near λ = 1 (decoupled state). As expected, in some cases, solvent water molecules were found to be extremely close to one or more solute atoms. When the energies of these configurations were re-evaluated at or near λ = 0, some yielded unrealistically large *negative* energies. Consequently, these samples were falsely assigned disproportionately high weights in the MBAR analysis, thus responsible for the erroneous ASFEs. A detailed example is provided in the SI. We, therefore, used the BAR estimator as implemented in pymbar to compute the free energy differences between neighboring λ-states and then summed up the pairwise free energy differences. Any *ΔG* obtained in this manner was not sensitive to the way the λ-dependent distance offset was added; see SI.

*Cycle Closure*. The cycle closure errors of 0.0 and −0.2 kcal/mol in Table 1, respectively, can be considered zero given the uncertainty of 0.2–0.4 kcal/mol. Most of the uncertainty arises from the ASFE steps. Nevertheless, the individual uncertainties of 0.1 to 0.4 kcal/mol for ASFEs are in line with what one would expect for force field calculations using a similar protocol (three repeats, < 1 ns simulation length per λ-state). The low cycle closure errors, together with the agreement between absolute solvation free energies for water and phenol using the four distance-shifting schemes (eqs 1a–1d), see SI, demonstrate the correctness of our approach.

**Table 1:** Cycle Closure Calculations for the ASFE and RSFE Differences of Two Tautomer Pairs, tp1516 and tp558^a^

	tp1516	tp558
*ΔG*_*solv*_(*T*1)	–7.2 ± 0.4	–9.4 ± 0.2
*ΔG*_*solv*_(*T*2)	–10.0 ± 0.1	–15.3 ± 0.1
*ΔG*_*gas*_(*T*1 → *T*2)	–0.9 ± 0.0	+4.0 ± 0.1
*ΔG*_*aq*_(*T*1 → *T*2)	–3.7 ± 0.0	–1.7 ± 0.1
		
cycle closure	**0.0** ± 0.4	**-0.2** ± 0.2

a See Appendix, Figure 3. All results are in kcal/mol. The free energy differences are reported as mean ± standard deviation of the total free energies differences (for the absolute or relative transformations) obtained from three independent replicates. Cycle closure is calculated as *ΔG*_*gas*_(*T*1 → *T*2) + *ΔG*_*solv*_(*T*2) – *ΔG*_*aq*_(*T*1 → *T*2) – *ΔG*_*solv*_(*T*1). The absolute legs were obtained with the decoupling method discussed in this work, the relative legs with the procedure described in the Appendix and in ref 34. The error estimate for the cycle closure was obtained by Gaussian error propagation from the individual standard deviations.

*First Solvation Free Energies*. For the tautomer pair used to study cycle closure error, no solvation free energies are known. In Table 2 we, therefore, present *ΔG*_*solv*_ results for 6 small solutes, plus the hydration free energy of water. We report results for MACE-OFF23­(S) and MACE-OFF23­(M). The MACE-OFF23­(S) results are in quite good agreement with experiment. The largest deviation from the experimental ASFE is obtained for phenol (0.6 kcal/mol). Of course, the number of systems studied is far too low to draw conclusions how the model would perform on larger and chemically more diverse solutes. Somewhat surprisingly, the results with the small model are in better agreement with the experimental values than those for the medium model; for MACE-OFF23­(M) all *ΔG*_*solv*_ results are too positive by 2–3 kcal/mol. However, the RSFE difference between ethane and methanol of – 7.9 kcal/mol obtained with MACE-OFF23­(M) has the correct order of magnitude, and ethanol is slightly less hydrophilic than methanol. In our recent work studying the properties of neat liquids, none of the NNPs considered reproduced condensed phase properties well.^33^ The performance of MACE-OFF23­(M), therefore, underlines that further refinements of NNPs may be required before relying on them for the prediction of condensed phase properties, including free energy differences.

**Table 2:** Solvation Free Energies *ΔG*_*solv*_ Reported as Mean ± Standard Deviation of the Total Absolute Solvation Free Energies Obtained from Three Independent Replicates^a^

	MACE-OFF23(S)	MACE-OFF23(M)	Experiment
Water	–6.5 ± 0.1		–6.3
Methane	+2.0 ± 0.0		+2.3
Ethane	+2.3 ± 0.1	+4.9 ± 0.1	+1.8
Methanol	–4.8 ± 0.1	–3.0 ± 0.1	–5.1
Ethanol	–4.3 ± 0.1	–2.2 ± 0.3	–5.0
Toluene	–1.3 ± 0.1		–0.9
Phenol	–6.0 ± 0.1		–6.6

a All results are in kcal/mol. The experimental values are taken from the FreeSolv^1^ or the Minnesota solvation database.^41^

*Summary and Outlook*. Masking and decoupling of atoms and molecules is a key step for alchemical transformations. The neighbor list, the common denominator of all NNP architectures currently in use, provides a suitable hook to achieve both tasks. In this work, we focused on absolute transformations; i.e., the decoupling of atoms. The relative free energy calculations carried out to test for cycle closure (see the Appendix and ref 34) used only masking. For relative transformations, in which larger groups of atoms need to be made to appear/disappear, e.g., the transformation of a hydrogen atom into an ethyl-group, it will be necessary to combine masking and decoupling to avoid end state problems.

These are first results, and the robustness and stability of the approach outlined here require further validation. Simulations in which all interactions are described by NNPs are occasionally unstable even without alchemical modifications. E.g., in the tautomer calculations, we had to apply flat-bottom harmonic well restraints to prevent the tautomeric hydrogen from flying away, even at the physical end points.^34^ Only a cursory attempt was made to optimize the λ-scheduling, which clearly depends on the scheduling of how the 4D-offset is added.

Not too surprisingly, the agreement with the experimental solvation free energies reported here is at best fair, with the MACE medium model resulting in worse results than the small model. Since current NNPs are mostly trained on single-molecule properties, their ability to reproduce condensed-phase properties is mediocre.^32,33^ FES connect the macroscopic, thermodynamic quantity (Δ)*ΔG* with the microscopic interactions between all atoms of the system;^42^ therefore, the agreement of, e.g., calculated ASFEs with the experimental data is a sensitive quality control for the method used to compute intra- and interatomic interactions. The neighbor list approach described here can be applied to any existing and future NNP architectures and training sets. Straightforward adaptations may be needed for NNPs in combination with classical long-range interactions and related approaches.^43^ ,^44^ The ability to carry out ASFE calculations with multiple NNPs using the same methodology will facilitate a more in-depth understanding of the respective strengths and weaknesses of NNPs.

## Supplementary Material



## Figures and Tables

**Figure 3:**
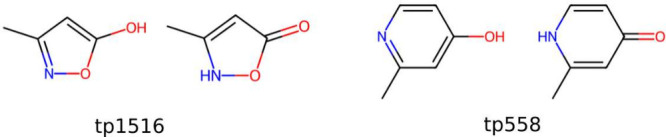
Two tautomer pairs used for calculating cycle closure.

## Data Availability

A collection of the scripts that were used to compute results presented here, as well as some input pdb files, can be found in the GitHub repository https://github.com/cbc-univie/asfe-4D/.

## Appendix

*Brief Description of the RSFE Calculations*. We calculated RSFEs with the pre-trained MACE-OFF23­(S) model^16^ in single precision for the two tautomer pairs shown in Figure 3. These are two of the model systems used in ref 34, where we provide the detailed description of our RSFE methodology for all-NNP simulations. We summarize the protocol used below; for full details, please refer to ref 34.

We employed a single-coordinate, dual-topology approach to carry out alchemical transformations between the two tautomeric states (*T*1, *T*2). Specifically, we used a hybrid structure that contained both keto and enol tautomeric hydrogens. Masking one of the hydrogens (i.e., treating it as a dummy atom) yields the desired tautomeric state. As outlined earlier, this was implemented by removing all pairs containing the atom to be masked from the neighborlist. We denote the energies of the simulation systems corresponding to *T*1 and *T*2, by masking the respective “superfluous” hydrogen, as *U*_1_ and *U*_2_, respectively. The energy of an intermediate *λ*-state is derived by linear interpolation between the two energy states with coupling parameter *λ*:

2
$$U\left(\lambda \right)=\left(1-\lambda \right)\cdot U_{1}+\lambda \cdot U_{2}+U_{restr}$$

The term *U*_*restr*_ in eq 2 contains several restraint terms: (1) *λ*-dependent harmonic bond and angle restraints were applied between the dummy atom and neighboring heavy atoms, except for the respective physical end state, to keep the dummy atom near its neighboring heavy atom.^45^ (2) Additionally, *λ*-independent flat-bottom harmonic well (FBHW) restraints were used in all *λ*-states to prevent occasional deprotonation of the tautomeric oxygen or nitrogen atom. These *λ*-independent FBHW restraints were used in all tautomer calculations, where deprotonations occurred frequently. FBHW restraints were not applied in the ASFE simulations of the small solutes (Table 2). Simulations along the alchemical paths (*T*1 → *T*2) were carried out at 11 alchemical *λ*-states, *λ* = 0.0, 0.05, 0.1, 0.2, 0.3, 0.5, 0.7, 0.8, 0.9, 0.95, 1.0, in both, the gas phase and in a cubic box of water. The initial side-length of water boxes was 21 Å. To account for equilibration, the first 200 ps of each 1.2 ns simulation per *λ*-state were discarded. Room temperature and pressure were maintained by Langevin dynamics and the Monte Carlo barostat. The time-step was 1 fs, except for simulations showing instabilities, where it was reduced to 0.5 fs. A total of 500 coordinate sets per *λ*-state were used to compute the free energy difference using MBAR, as implemented in pymbar. Each relative free energy difference in the gas phase (*ΔG* ^*gas*^(T1 → *T*2)) and in solution (*ΔG* ^*aq*^(T1 → *T*2)) is the mean of three repeats; the standard deviation of the three individual results was used as the error estimate.

*Modifications of the ASFE Protocol*. ASFE calculations of the tautomers, for the most part, were carried out identically as described in the Simulation Details section of the main manuscript. Minor modifications were introduced to align the protocol with the relative approach described above and/or to improve accuracy. All simulations were carried out in ≈21 Å water boxes. FBHW distance restraints were applied between the tautomeric hydrogen and its corresponding heavy atom in all *λ*-states. To improve overlap, particularly in the range between *λ* = 0.2 and 0.5, we used the following 18 *λ*-values: 0.0, 0.0048, 0.0192, 0.0433, 0.0769, 0.1202, 0.1731, 0.2356, 0.2716, 0.3077, 0.3486, 0.3894, 0.4351, 0.4808, 0.5817, 0.6923, 0.8125, 1.0. Any unstable simulations were repeated with a 0.5 fs (instead of a 1 fs) time-step.

## Notes

Details on how the distance-shifting functions and set of λ values were chosen, additional information on the production simulation settings, and some further notes on MBAR high weight contributions (PDF)
