# Supplementary material for: Architecture-Independent Absolute Solvation Free Energy Calculations with Neural Network Potentials
Source: J Phys Chem Lett. 2025 Nov 11;16(46):12080–6. doi: 10.1021/acs.jpclett.5c02980 (PMC12641469; doi:10.1021/acs.jpclett.5c02980)
Supplement: Supplementary file 1 [file jz5c02980_si_001.pdf]

Supporting information – Architecture independent absolute solvation free energy calculations with neural network potentials

Anna Katharina Picha,[†,‡] Sara Tkaczyk,[¶,§] Thierry Langer,[¶] Marcus Wieder,[*,‖] and Stefan Boresch[*,†]

†*University of Vienna, Faculty of Chemistry, Institute of Computational Biological Chemistry, Austria*

‡*University of Vienna, Vienna Doctoral School of Chemistry (DosChem), Austria*

¶*Department of Pharmaceutical Sciences, Pharmaceutical Chemistry Division, Josef-Holaubek-Platz 2, University of Vienna, 1090 Vienna, Austria*

§*Vienna Doctoral School of Pharmaceutical, Nutritional and Sport Sciences (PhaNuSpo), University of Vienna, Vienna, Austria*

‖*Open Molecular Software Foundation, Davis, California 95616, United States*

E-mail: marcus.wieder@gmail.com; stefan.boresch@univie.ac.at

Selecting and optimizing the shifting scheme

Shifting on single frames

We first evaluated the four shifting schemes (Equations 1a–1d in the main manuscript) by performing potential energy scans as a function of the shifting parameter λ, using 1000 coordinate frames sampled from an equilibrium simulation of one ethane molecule in a box with

249 water molecules. The reference frames were obtained from the unperturbed system at $\lambda = 0$ (i.e., native solute–solvent interactions). For each frame, all solute–solvent interatomic distances d_{ij} within the cutoff radius ($d_{ij} < r_{cut}$) were modified according to the four shifting functions (Equations 1a–1d in the main manuscript), resulting in four distinct data sets:

$$\{(\lambda, d_{ij}^{(k)}(\lambda)) \mid \lambda = \frac{i}{100}, i = 0, \ldots, 100\}, \quad k \in \{1, 2, 3, 4\}.$$

The potential energy was evaluated for each shifted configuration and recorded. The results are presented in Figure 1; from each potential energy value the corresponding $\lambda = 1$ energy (i.e., no solute–solvent interactions) was subtracted.

In Figure 1, yellow regions indicate values close to zero, indicating the regime where all solute–solvent interactions have effectively been switched off. One clearly sees that the linear shifting function suppresses solute–solvent interactions already around $\lambda = 0.4$. In the 4D shifting scheme, this happens around $\lambda = 0.7$. Both "to cutoff" shifting schemes extend this even further, with interactions vanishing only for $\lambda > 0.8$.

Solvation free energy differences for two test systems

To further investigate the efficiency of the shifting functions and λ-scheduling schemes, we calculated absolute solvation free energies (ASFEs) for water and phenol for all four shifting options. All calculations were carried out using MACE-OFF23(S) in single precision. For each function, three independent runs were performed for both test systems, employing 20 equidistant λ values ($\lambda \in \frac{i}{19}, i = 0, \ldots, 19$). For phenol, the solvation free energy calculations were initiated from a simulation box containing 249 water molecules with an average side length of 18.8 Å. For water, the system consisted of 254 water molecules with an average side length of approximately 18.9–19 Å. The time step in all simulations was 1 fs.

The resulting pairwise free energy differences between neighboring λ-states, obtained with the BAR algorithm, are shown in Figure 2, and the ASFEs are reported in Table 1.

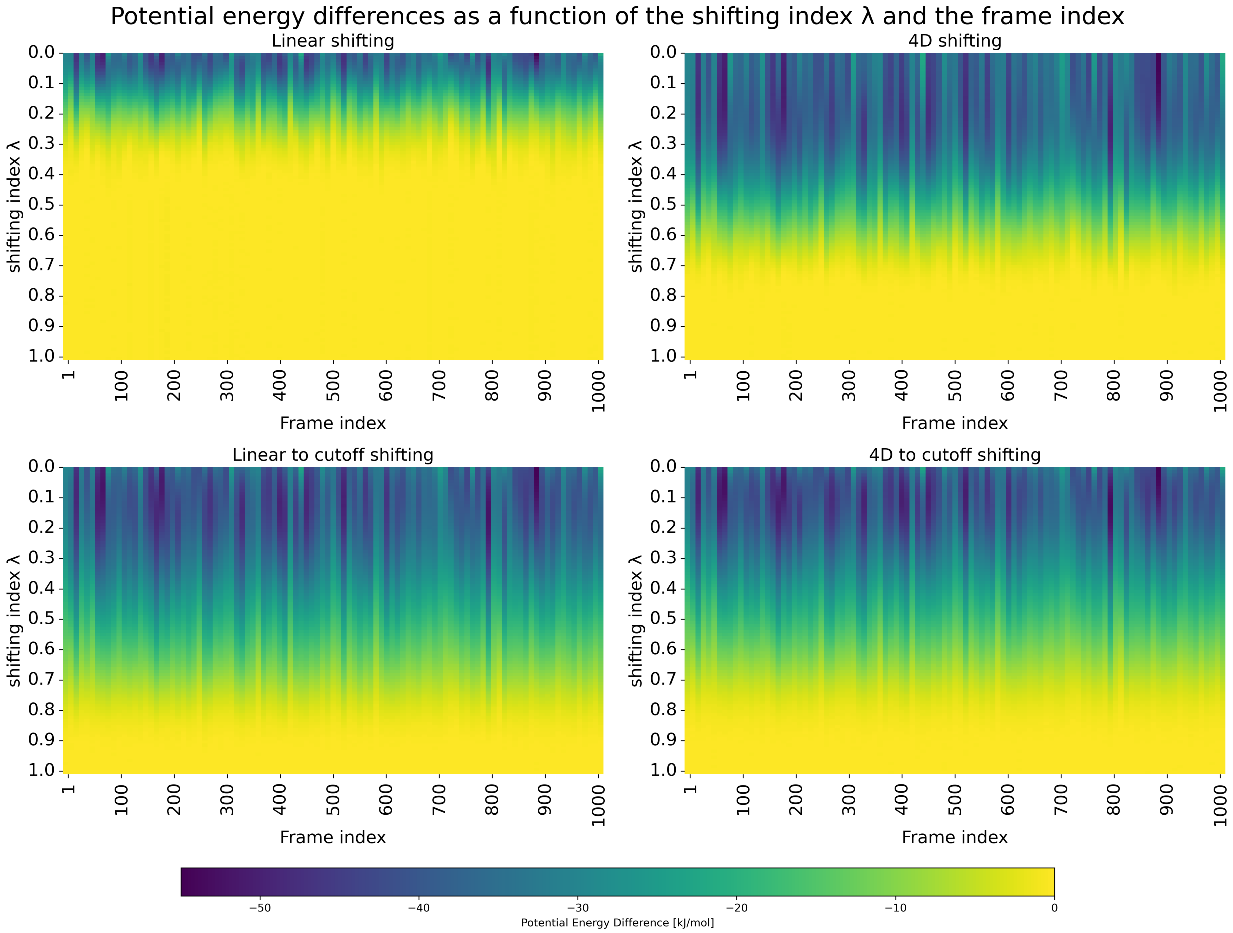

Figure 1: Potential energy differences as a function of the shifting parameter λ for 1000 samples of ethane in water for each of the four shifting schemes. From each potential energy value, the corresponding $\lambda = 1$ energy (i.e., no solute–solvent interactions) was subtracted. That is, values close to zero indicate the regime where all solute–solvent interactions have effectively been switched off.

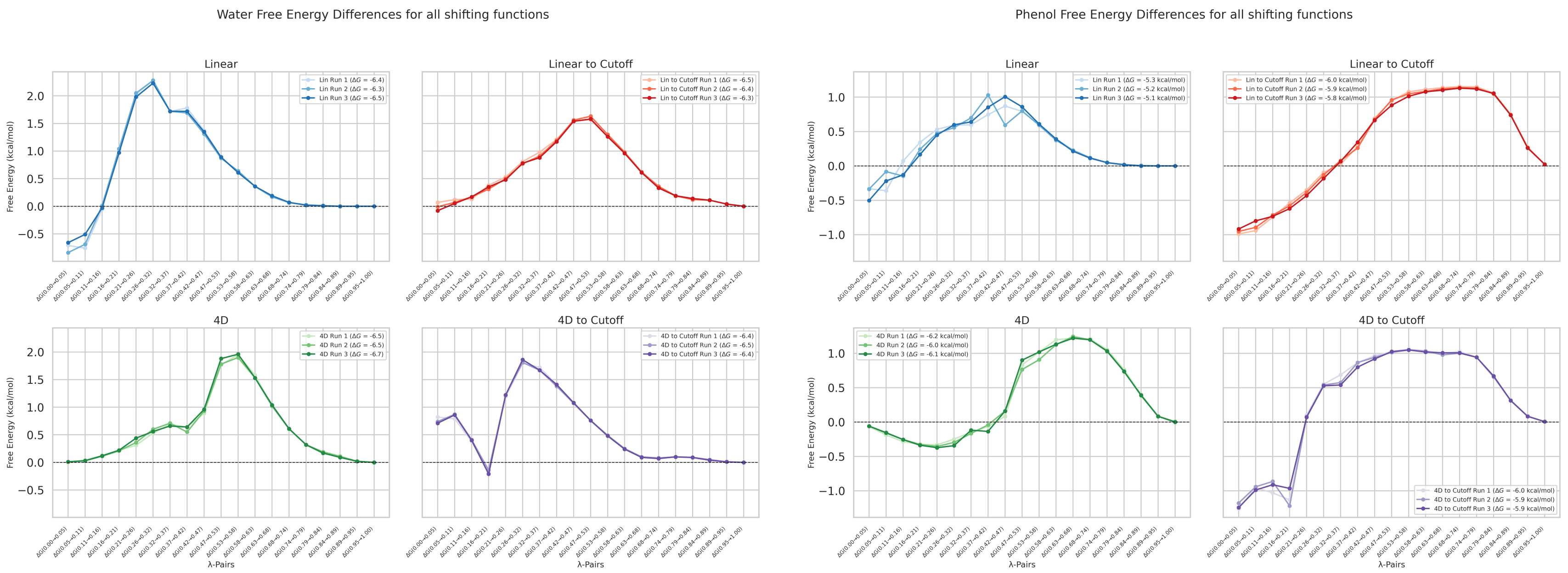

Figure 2: $\Delta G(\lambda_i \to \lambda_{i+1})$ obtained with BAR for λ pairs for all four shifting functions for (left) water and (right) phenol.

As shown in Table 1, all four shifting schemes yield ASFEs for water that agree within statistical uncertainty. For phenol, the ASFE obtained with the linear shifting scheme deviates somewhat from the other methods, which lead to consistent results, as they should. Both Figure 1 and Figure 2 indicate that in the case of linear shifting most contributions arise for small λ-values. We, therefore, performed additional simulations for phenol using linear shifting, focusing on the first half of the shifting region: $\lambda \in \{0.0167, 0.0333, 0.0800, 0.1350, 0.1850, 0.2350, 0.2900, 0.3450, 0.3950, 0.4450, 0.5000, 0.5550\}$. Reevaluation of the ASFE with all 32 λ-states yielded $\Delta G = -6.1 \pm 0.8$ kcal/mol, indicating that with a denser sampling of the initial λ range, the linear shifting function produces results comparable to the other schemes. The relatively large statistical error and the need for 32 λ-states, however, indicates that linear shifting should not be used, while the three other schemes performed similarly. Since the linear-to-cutoff scheme worked at least as good as both 4D variants, we decided to use it for all production calculations reported in the main manuscript.

Table 1: Comparison of all four shifting schemes for water and phenol. All ASFE values are in kcal/mol.

solute	linear	linear-to-cutoff	4D	4D-to-cutoff	experiment
water	-6.4 ± 0.1	-6.4 ± 0.1	-6.6 ± 0.1	-6.5 ± 0.0	-6.3
phenol	-5.2 ± 0.1	-5.9 ± 0.1	-6.1 ± 0.1	-5.9 ± 0.1	-6.6

Further optimization of the production protocol

To improve computational efficiency of the linear-to-cutoff shifting scheme, we reduced the number of λ values by re-evaluating the free energy differences with the BAR algorithm on various subsets of the initial 20 equidistant states. The analysis showed that removing points in the interval $[0, 0.5]$ increased statistical uncertainty, whereas a reduction in the interval $[0.5, 1]$ had little effect. Consequently, we selected a final set of 15 λ values, obtained by removing five points from $[0.5, 1]$, resulting in an asymmetric distribution that preserves accuracy while lowering computational cost: $\lambda \in \{\frac{i}{19} \,|\, i = 0, 1, 2, 3, 4, 5, 6, 7, 8, 9, 11, 13, 15, 17, 19\}$.

The ASFEs using the linear-to-cutoff scheme and these 15 λ-values of -6.4 ± 0.1 kcal/mol for water and -5.9 ± 0.1 kcal/mol for phenol are identical to those reported in Table 1. Thus, the reduced λ schedule provided results consistent with the full protocol for both test systems.

Additional details of the production simulation settings

Table 2: Box sizes after 100ps equilibration with the MACE-OFF23(S) model for small solute ASFE calculations.

Solute	# water molecules	Box size[a]
Water	437	22.7 Å
Methane	444	23.0 Å
Ethane	441	22.8 Å
Methanol	443	22.8 Å
Ethanol	442	22.9 Å
Toluene	439	23.0 Å
Phenol	440	22.9 Å

[a]Instantaneous boxsize after 100 ps equilibration

MBAR: spurious high weight contributions

We investigated why the MBAR algorithm[1] fails to produce meaningful results and identified several problematic configurations. In all cases investigated, solvent waters were extremely close to one or more solute atoms, as is to be expected near the decoupled end state, i.e., when most solute–solvent interactions have been turned off. When reevaluting the energies of these configurations at or near $\lambda = 0$ (interacting initial state), in some cases one obtains extremely negative energies. These samples were consequently assigned disproportionately high weights in the MBAR algorithm. Such configurations, however, should be highly repulsive, resulting in large, positive energies and thus negligible weights. Thus, the erroneously negative energies for these overlapping configurations (with some atoms almost on top of each other) are

responsible for incorrect ASFEs when attempting to use MBAR.

As a specific example, in the phenol system, we observed a configuration sampled at $\lambda = 1$ where the distance between a solute and a solvent atom is only 0.157 Å, corresponding to atomic overlap. When re-evaluated at $\lambda = 0$, this configuration produced a nonphysical energy, even more negative than, e.g., some non-overlapping configurations sampled at $\lambda = 0$, instead of the expected strong repulsion. It is likely that this artifact originates from a lack of training data. Retraining as described by Moore et al.[2] may avoid this issue.

References

(1) Shirts, M. R.; Chodera, J. D. Statistically optimal analysis of samples from multiple equilibrium states. *The Journal of Chemical Physics* **2008**, *129*, 124105.

(2) Moore, J. H.; Cole, D. J.; Csanyi, G. Computing hydration free energies of small molecules with first principles accuracy. *arXiv* **2024**,
